# Novel Medicine for Endometriosis and Its Therapeutic Effect in a Mouse Model

**DOI:** 10.3390/biomedicines8120619

**Published:** 2020-12-16

**Authors:** Young Sang Kim, Yu Jin Kim, Myung Joo Kim, Sang Jin Lee, Hwang Kwon, Jae Ho Lee

**Affiliations:** 1 CHA Fertility Center Seoul Station, Seoul 04637, Korea; p0892@chamc.co.kr (Y.S.K.); splower2@cha.ac.kr (M.J.K.); 2 Laboratory of Reproductive and Molecular Medicine, CHA Fertility Center Seoul Station, Seoul 04637, Korea; yj_kim@chamc.co.kr; 3 Institute of Animal Genetic Resources Affiliated with Traditional Hanwoo Co., Ltd., Boryeong 33402, Korea; leesj914215@naver.com; 4 Department of Obstetrics and Gynecology, Fertility Center of CHA Bundang Medical Center, CHA University School of Medicine, Seongnam 13496, Korea; hkwon@chamc.co.kr; 5 Department of Biomedical Science, College of Life Science, CHA University, Pocheon 11160, Korea

**Keywords:** endometriosis, animal model, Dienogest, Cabergoline, therapeutic effect, combined medications

## Abstract

Current therapeutic medicines for endometriosis cannot be administered during assisted reproductive technology (ART) because they have bad effects during pregnancy. In this study, we created an animal model of endometriosis and evaluated the therapeutic effect of progestin (Dienogest), dopamine agonist (Cabergoline), and their combination (Dienogest + Cabergoline). We established a mouse model mimicking human endometriosis. The mice with endometriosis were then treated with a single drug (Dienogest or Cabergoline) or both drugs (Dienogest + Cabergoline) for 14 days. An immunohistological study was then performed to analyze inflammatory lesions in the recipient mice. Real-time polymerase chain reaction (RT-PCR) and Western blotting were also performed to determine the levels of genes and proteins in inflammatory lesions to assess the recovery of endometriosis. Histologic staining showed that all medication groups showed a clear decrease in the inflammatory phenotype in the uterus, peritoneum, and intestine. Gene and protein expression analysis showed a therapeutic effect in all medication groups. In conclusion, Cabergoline had a therapeutic effect similar to that of Dienogest and could be used as an alternative to Dienogest during ART for patients with infertility; compared to the individual drugs, the combination treatment has a synergistic effect on endometriosis.

## 1. Introduction

Endometriosis is a common gynecological disease that is defined as the presence of endometrial tissue outside the uterus. Endometriosis is often seen as a reproductive disease. It can impact the ovaries, uterus, and fallopian tubes, causing pelvic pain and subfertility [1,2]. Current approaches for treating endometriosis include pharmacological therapy and surgical removal of endometriotic lesions [3,4,5,6,7,8,9]. Classical pharmacological therapies primarily aim to suppress endogenous estrogen production with oral contraceptives, gonadotropin-releasing hormone (GnRH) agonists, and androgenic agents or aromatase inhibitors [9,10,11,12,13,14]. Elagolix, a new oral nonpeptide GnRH antagonist that can partially or fully suppress estrogen, has shown efficacy in controlling both dysmenorrhea and chronic pelvic pain [15]. However, these medications are associated with substantial side effects, which limit their prolonged usage. In addition, endometriosis is likely to recur following treatment cessation. Moreover, these medications cannot be administered during assisted reproductive technology (ART) or in pregnant women because they have bad effects during pregnancy. Surgical removal of endometriotic lesions is only temporarily effective. In addition, this approach is associated with a high recurrence rate [5,6,16]. Other treatment options of endometriosis like phytotherapy might be a promising alternative and complementary strategy [17].

Regarding the mechanism of endometriosis-related pelvic pain, the presence of nerve fibers in peritoneal endometriotic lesions and the eutopic endometrium in women with endometriosis has received much attention recently [18,19,20]. Endometriotic lesions innervated by nerve fibers colocalize with immature blood vessels, suggesting that they can cause pelvic pain and local tenderness [21]. Some current treatments for endometriosis, such as combinations of oral contraceptives and progestogens, can significantly decrease nerve fiber density in the endometrium and myometrium in women with endometriosis [22,23]. Novel treatment strategies for endometriosis should guarantee the long-term cure of affected patients. For this purpose, key processes in the pathogenesis of endometriosis have been identified, and these processes might serve as potential therapeutic targets [24,25]. One of these key processes is angiogenesis. New vessel formation has long been recognized as a feature of endometriosis [10,26,27]. Similar to tumors and their metastases, the survival of endometriotic lesions is crucially dependent on the establishment of an adequate blood supply. Moreover, in peritoneal fluid from patients with endometriosis, the concentrations of various angiogenic growth factors are high, while the concentrations of antiangiogenic compounds are decreased [10,28,29]. Agents that can inhibit this mechanism include endogenous angiogenesis inhibitors (e.g., Endostatin) [10], cyclooxygenase-2 (COX-2) inhibitors (e.g., Rofecoxib and Celecoxib) [30], dopamine agonists (e.g., Cabergoline), and progestins (e.g., Dienogest). Among these drugs that inhibit angiogenesis, Dienogest and Cabergoline [31] are commonly prescribed for outpatient treatment. There is a need to find drugs for endometriosis treatment that do not inhibit ovulation and can be administered during pregnancy. The best combination of these drugs also needs to be found to achieve the maximum treatment effect [24].

Dienogest, a progestin and a 19-nortestosterone derivative, has good oral bioavailability. It is highly selective for progesterone receptors. It is suitable for long-term applications because it does not induce hypoestrogenism like GnRH agonists [31]. Thus, Dienogest is commonly used to treat endometriosis due to its strong endometrial efficacy. It has antiproliferative and anti-inflammatory effects on endometriotic lesions [32]. In addition, it can significantly decrease nerve fiber density in the endometrium and myometrium of women with endometriosis [33]. There is evidence that dopamine agonists are effective in preventing ovarian hyperstimulation syndrome in animals and humans [34,35]. Their activation is involved in the regulation of angiogenic events. They can even block tumor-related angiogenesis and vascular permeability by interfering with vascular endothelial growth factor signaling [36]. A randomized experimental study showed the effects of two well-known dopamine agonists (Bromocriptine and Cabergoline) in a rat endometriosis model in comparison with GnRH analogs and controls [37]. The aim of this study was to establish an animal model of endometriosis and evaluate the therapeutic effect of novel medicines on endometriosis using this animal model.

## 2. Experimental Section

### 2.1. Animals

**Figure 1:**
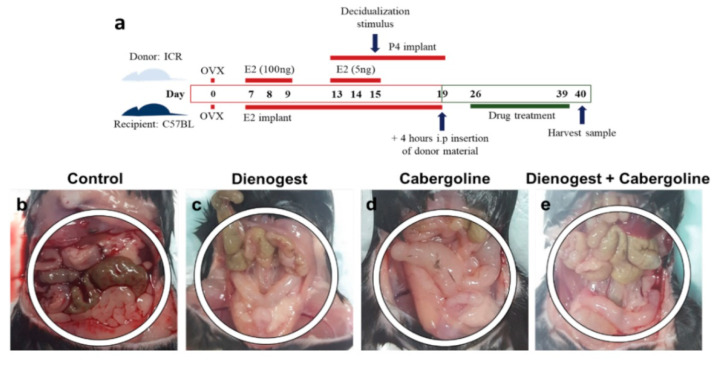
Schematics showing procedures for preparing for the endometriosis animal model. (**a**) Whole procedure for inducing endometriosis with OVX (ovariectomy), E2 (estradiol), and P4 (progesterone) treatment. (**b**–**e**) Image of the abdomen area during the anatomic procedure used to establish the endometriosis animal model; (**b**) Control, (**c**) Dienogest, (**d**) Cabergoline, and (**e**) combined treatment.

We generated an animal model of endometriosis with C57BL/6 mice as recipients and ICR mice as donors (both approximately 8 weeks old, 20~25 g). We established the endometriosis animal model following a published study [38] with modifications (Figure 1a). All experimental procedures for animal breeding and care complied with the Institutional Animal Care and Use Committee (IACUC: SYUIACUC 2011-020, 30 December 2018) regulations of Samyook University. Outbred white ICR mice and inbred black C57BL/6 mice were purchased from Oriental Bio (Sungnam, South Korea). They were maintained at 23 °C with 12 h light/12 h dark in an animal facility.

### 2.2. In Vivo Model of Endometriosis

First, we performed surgical ovariectomy (day 0) on both donor mice and recipient mice before estrogen priming. The donor mice were primed with 100 ng of estradiol-17β (day 7 to day 9) by subcutaneous injection. They were continuously treated with progesterone delivered via P4 implants (P4-M, Belma Technology, Belgium) from day 13 to day 19. Finally, they were injected with 5 ng of estradiol-17β in sesame oil on days 13, 14, and 15. Decidualization was then induced in one uterine horn using 20 µL of oil on day 15. Endometrial tissue in the process of being shed from the decidualized horn was recovered from a mouse killed on day 19 at 4 h after P4 withdrawal (removal of the implant) by opening the horn longitudinally in a Petri dish and scraping the tissue away from the myometrial layer using a scalpel. On day 19, endometrial tissue fragments extracted from donor mice were dissolved in saline and injected into the pelvic cavity of anesthetized recipient mice (approximately 40 mg of tissue/0.2 mL of phosphate-buffered saline (PBS) per mouse) prepared with E2 (estradiol-17β) implants (E2L-M, Belma Technology, Belgium) from day 7. The recipient mice were divided into four groups (5 mice per group): (1) control (injected with only saline); (2) D, Dienogest (1 mg/kg) treatment; (3) C, Cabergoline (0.05 mg/kg) treatment; and (4) D + C, Dienogest (1 mg/kg) + Cabergoline (0.05 mg/kg) treatment. The recipient mice were treated with a single drug (Dienogest or Cabergoline) or combined drugs (Dienogest + Cabergoline) for 14 days (day 26 to day 39). Each medication dose was recalculated for mouse body weight from the original human body weight doses. All experiments were repeated three times. Thus, the total number of mice was 15 per group (total of 60 mice) for statistical analysis. On day 40, peritoneum, uterus, and intestinal tissue samples were collected. Histological samples were fixed in 4% paraformaldehyde (PFA) and then kept at 4 °C. For gene and protein expression analysis, samples were stored in a −80 °C deep freezer until the experiment.

### 2.3. Histological Study

Peritoneum, uterus, and intestinal tissue samples were collected and subjected to histological hematoxylin and eosin (H&E) staining to analyze inflammatory lesions from recipient mice following routine protocols. Each tissue sample was fixed in 4% paraformaldehyde fixative for 30 min at room temperature. These fixed tissue samples were dehydrated and embedded in paraffin. The paraffin-embedded sample block was then serially cut (cross-sectioned at a 5-µm thickness) using a rotary microtome (Leica RM 2135, Leica Instruments, Nussloch, Germany). The cross-sections were deparaffinized in xylene, rehydrated through a graded ethanol series, and subjected to H&E staining with routine methods. The stained samples were then observed and imaged with an inverted light microscope (Eclipse Ti-U, Nikon, Tokyo, Japan).

### 2.4. Immunohistochemistry Study

Immunohistochemistry was performed on the collected mouse tissue samples. A mouse or rabbit-specific Diaminobenzidine (DAB) (ABC) Detection IHC Kit (ab64259, ab236469, Abcam, Cambridge, UK) was used following the manufacturer’s protocols. Each tissue slide was deparaffinized with xylene for 10 min twice and rehydrated with a graded EtOH series (100%, 95%, 80%, and 75%) for 10 min each at room temperature. For antigen retrieval, the samples were boiled in 0.01 M citrate buffer (pH 6.0) for 20 min in autoclavable jars (Nalgene Jars). The samples were then permeabilized with PBS containing 0.1% Triton-X for 20 min at room temperature and washed with fresh PBS solution three times. Subsequently, the slides were stained with a mouse anti-estrogen receptor (ER)-α antibody (SC-8002, monoclonal, Santa Cruz Biotechnology, Dallas, TX, USA), mouse anti-ER-β antibody (SC-390243, monoclonal, Santa Cruz Biotechnology, Dallas, TX, USA), and mouse monoclonal anti-nerve growth factor (NGF) antibody (11050-RP02, Sino Biological, Beijing, China) in a humidified chamber overnight at 4 °C. Antibodies were diluted 1:100 in PBS containing 2.5% bovine serum albumin (BSA). After washing twice in PBS, the slides were incubated with biotinylated conjugated goat anti-mouse or anti-rabbit secondary antibody for 1 h at room temperature. They were then incubated with streptavidin peroxidase for 10 min at room temperature. After that, the slides were treated with a mixture of 20 µL of DAB chromogen and 1 mL of DAB substrate solution for 10 min at room temperature. In the final step, each sample was counterstained with hematoxylin solution (ab220365; Abcam, Cambridge, UK) for 5 min at room temperature. Each stained sample was then washed with PBS, added to mounting medium for IHC (Ab64230, Abcam, Cambridge, UK), and covered with a cover glass. Light microscopy images of stained tissues were acquired using an inverted microscope equipped with a DS-5i camera (Eclipse Ti-U, Nikon, Japan). Each sample image was analyzed as a single picture (TIFF format) using NIS Elements viewer imaging software version 4.6 (Nikon, Tokyo, Japan).

### 2.5. RT-PCR and Real-Time qPCR for Gene Expression Analysis

To analyze the therapeutic effect on endometriosis at the molecular level, RT-qPCR was performed to determine the expression levels of Er-α, Er-β, Chemokine ligand (Ccl)2, Ccl5, and interleukin (Il)-6. First, we used 1 mL of TRIzol (15596026, Thermo Fisher Scientific^TM^, Carlsbad, CA, USA) to extract total RNA from each sample according to the manufacturer’s instructions. Next, the isolated RNA concentration was analyzed using a NanoDrop (Thermo Fisher Scientific™, Waltham, MA, USA). Total RNA was reverse transcribed to cDNA using AccuPower^®^ CycleScript RT PreMix (Bioneer, Daejeon, Korea) and then amplified with AccuPower^®^ Taq PCR PreMix (Bioneer, Daejeon, Korea) using primer sets specific for Er-α, Er-β, Ccl2, Ccl5, and Il-6, with glyceraldehyde-3-phosphate dehydrogenase (Gapdh) as a housekeeping gene for normalization. The primers are listed in Table 1. PCR cycles were run as following: 1 min at 95 ℃, then denaturation for 30 s at 95 ℃, annealing for 30 s at 60 ℃, and extension for 50 s at 72 ℃ followed by 30 cycles. The PCR products were resolved on 2% agarose gels with Safeview™ DNA stain (G108, Applied Biological Materials, Richmond, BC, Canada). The gels were visualized under UV illumination using a gel documentation system (WSE-6100 LuminoGraph; ATTO, Tokyo, Japan). Finally, the intensity of the product band was analyzed using ImageJ software. Additionally, real-time qPCR was performed using SsoAdvanced Universal SYBR Green Supermix (Bio-Rad, Hercules, CA, USA) on a spectrofluorometric thermal cycler (CFX96 Touch Real-Time PCR Detection System, Bio-Rad). PCR cycles were run as following: 3 min at 95 ℃, then denaturation for 10 s at 95 ℃, annealing for 30 s at 60 ℃, and extension for 20 s at 72 ℃ followed by 40 cycles. The comparative threshold cycle (CT) method was used, and the level of PCR product for each gene was normalized against the corresponding level of the PCR product for Gapdh. Samples were evaluated in triplicate.

### 2.6. Western Blotting for Protein Expression Analysis

Protein profiling was performed for inflammatory lesions by Western blotting with ER-α, ER-β, CD61, and NGF antibodies to quantify the anti-endometriosis effect. Samples were homogenized with protein extraction buffer (PRO-PREPTM 17081, iNtRON, Sungnam, Korea). Protein extracts were boiled with 4× Laemmli buffer, resolved with 8% SDS–PAGE, and transferred to nitrocellulose membranes (1620097, Bio-Rad, Hercules, CA, USA). These membranes were incubated in blocking buffer (TBS with 0.1% Tween-20 and 5% BSA) at room temperature for 1 h and then incubated with the respective primary antibodies (anti-ER-α, anti-ER-β, anti-CD61, anti-NGF, and anti-β-actin antibodies diluted in blocking buffer) at 4 °C overnight. After washing four times with TBS containing 0.1% Tween-20 for 5 min each, membranes were incubated with a horseradish peroxidase-conjugated donkey anti-mouse or anti-rabbit IgG secondary antibody (5178-2504 and 5196-2504; Bio-Rad, Hercules, CA, USA). Immunoreactive bands were visualized using enhanced chemiluminescence (Clarity™ Western ECL Substrate 1705060; Bio-Rad, Hercules, CA, USA). Band images were acquired using a gel documentation system (WSE-6100 LuminoGraph; ATTO, Tokyo, Japan). For quantitative analysis, the captured image was analyzed with ImageJ software (NIH).

### 2.7. Statistical Analyses

All data are expressed as the means ± standard error of the mean (SEM) of triplicate measurements. Statistical analyses were carried out using two sample Student’s *t*-test to compare the medication in both groups (control and treated) and two-way ANOVA with significance level set at * *p* < 0.05, ** *p* < 0.01, and *** *p* < 0.001. Significant differences are indicated by asterisks (* *p* < 0.05, ** *p* < 0.01, and *** *p* < 0.001) in each figure.

## 3. Results

### 3.1. Gross Findings of the Pelvic Cavity

We successfully established a mouse model of human endometriosis using endometrial tissue fragments extracted from donor mice and injected into the pelvic cavity of recipient mice (Figure 1a). In terms of gross findings, the positive control showed a severe inflammatory lesion pattern in the pelvic cavity (Figure 1b). In total, 14 of 15 head mice among the control group showed an inflammatory lesion in the pelvic cavity. Therefore, endometrial tissue injected mice showed 93.33% endometriosis phenotype ratios lesion in the pelvic cavity (Table 2). Inflammation was observed evenly around the peritoneum, uterus, and intestine in the control group. However, in experimental groups D (Dienogest), C (Cabergoline), and D + C (combined treatment Dienogest + Cabergoline), almost all mice had no inflammatory lesions in the pelvic cavity. Experimental groups D, C, and D + C had mild edematous changes in the uterus. However, they showed no inflammatory changes. There was no significant difference in the treatment effect among groups D, C, and D + C (Figure 1c–e).

### 3.2. Histological Study of the Endometriosis Animal Model

**Figure 2:**
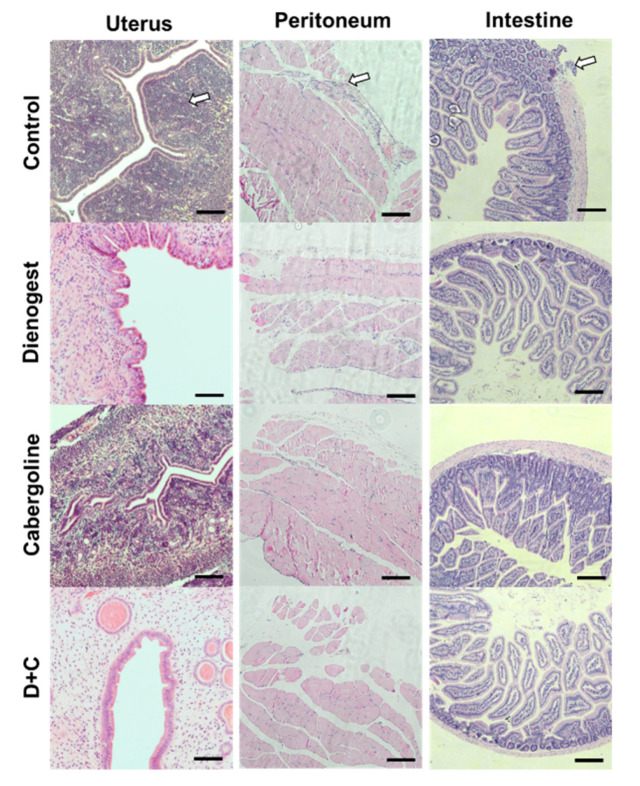
Histological images of the endometriosis control, Dienogest, Cabergoline, and combined treatment. The arrowhead is an endometriosis lesion; magnification, 200×; scale bar, 100 μm.

In the control group, the observed endometriosis phenotype included brown/black lesions (Figure 2). In H&E staining images, these lesions demonstrated evidence of hemosiderin and/or hemorrhage in inflammatory tissues in the epithelial layers of the uterus, peritoneum, and intestine. The histological study also showed a significantly reduced inflammatory phenotype in groups D, C, and D + C compared to the control. In particular, the inflammatory response seemed to be lower in group D + C than in groups D and C.

### 3.3. Immunohistochemistry Data

**Figure 3:**
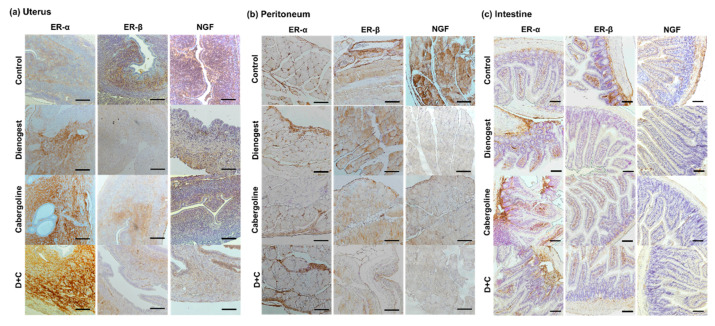
Light microscopy images of immunohistochemical analysis with anti-ER (estrogen receptor)-α, ER-β, and NGF (nerve growth factor) antibodies. (Magnification, 1000×). (**a**) Uterus, (**b**) peritoneum, and (**c**) intestine tissue. The results presented are the DAB-positive signal (brown) and hematoxylin (blue) for nuclear counterstaining; scale bar, 100 μm.

Next, to evaluate the effectiveness of pharmacotherapy, we examined the inflammatory protein expression pattern and localization for ER-α, ER-β, and NGF in the uterus, peritoneum, and intestine with immunohistochemistry staining (Figure 3). Most positive signals were localized in connective tissues, such as the extracellular matrix area and surface region. ER-β and NGF exhibited stronger positive staining in the control group than in the other groups. In contrast, ER-α showed stronger signals, while ER-β and NGF showed weaker staining in groups D, C, and D + C than in the control. In the case of ER-β and NGF, groups D and C showed the same staining intensity, while group D + C showed an obvious decrease compared with the control.

### 3.4. Gene Expression Analysis by RT-PCR and Real-Time qPCR

**Figure 4:**
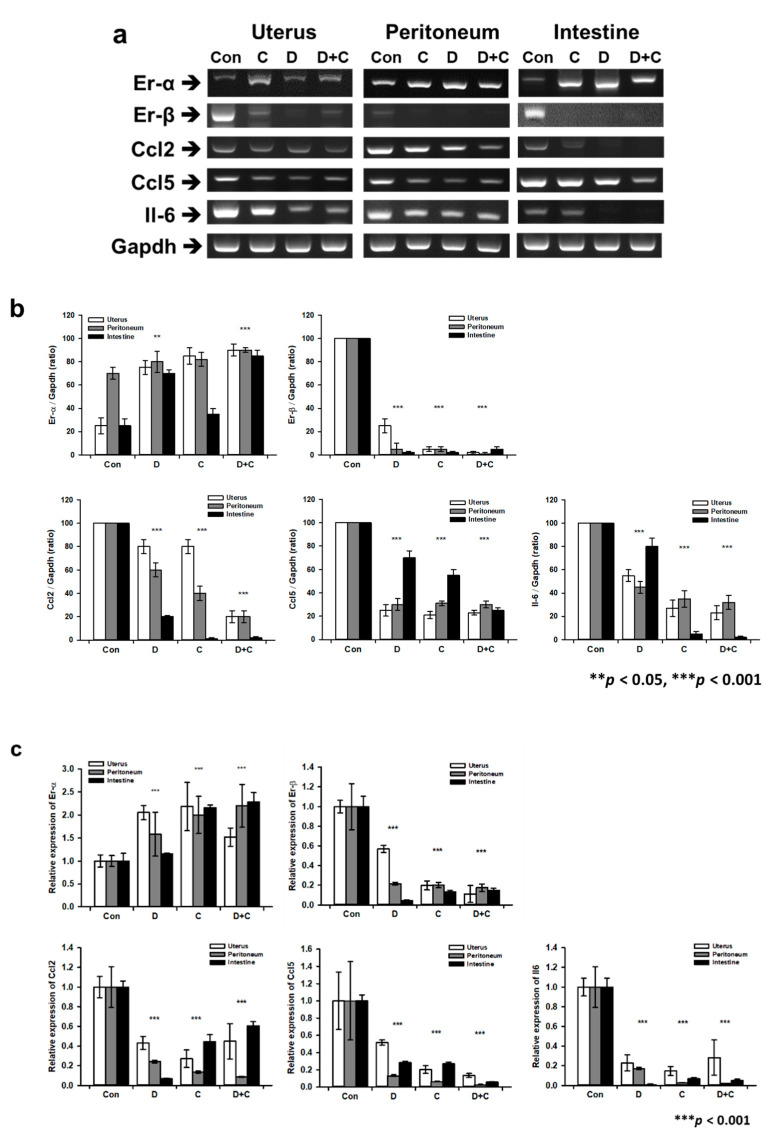
Analysis of gene expression in the inflammatory lesions of endometriosis tissue using RT-PCR (real-time polymerase chain reaction), (**a**) RT-PCR bands for Er-α, Er-β, Ccl (Chemokine ligand)2, Ccl5, and Il (Interleukin)-6 mRNA expression in the control and experimental groups (Dienogest, Cabergoline, and combined treatment). (**b**) Graph of RT-PCR product band intensity according to treatment in the experimental groups. (**c**) Graph of real-time qPCR with Er-α, Er-β, Ccl2, Ccl5, and Il-6 mRNA expression in the control and experimental groups (Dienogest, Cabergoline, and combined treatment). All experiments were repeated three times for statistical analysis (±SD, ** *p* < 0.01, and *** *p* < 0.001).

RT-PCR was performed to quantify gene expression levels in endometriosis. Based on gene expression levels in the endometriosis model, groups D, C, and D + C showed therapeutic efficacy for endometriosis tissue (Figure 4a,b). Er-α showed significantly increased expression in groups D and D + C except the C group (F 4.842, *p* < 0.05). In particular, the expression levels of Er-β were significantly decreased in the peritoneum, uterus, and intestine tissues of groups D, C, and D + C. All inflammation-related genes, such as Ccl2, Ccl5, and Il-6, showed significantly decreased expression in groups D, C, and D + C compared to the control. The inflammation-related gene expression profile showed that groups D and C had similar therapeutic effects. In groups D + C, the expression levels of Ccl2, Ccl5, and Il-6 were decreased more than those in groups D and C. In addition, real-time qPCR data exhibited a similar decreasing and increasing pattern compared with the band’s PCR products on agarose gels, with significantly decreased Er-β, Ccl2, Ccl5, and Il-6 levels in the treated groups. C and D + C showed a strong effect in the uterus, peritoneum, and intestine, and D exhibited a weak effect in the uterus compared with that of the other medicines. Er-α expression was significantly increased compared with that of the control group (F 4.401, *p* < 0.05). All of the drugs showed an approximately 2-fold increase in the uterus and peritoneum, respectively (Figure 4c). Therefore, the expression of inflammation-related genes, such as Ccl2 (F 9.873), Ccl5 (F 0.960), and Il-6 (F 2.098), significantly decreases compared with that of control group in the uterus, peritoneum, and intestine. Based on the gene expression levels, there was no synergistic effect of D + C.

### 3.5. Protein Expression Analysis by Western Blotting

**Figure 5:**
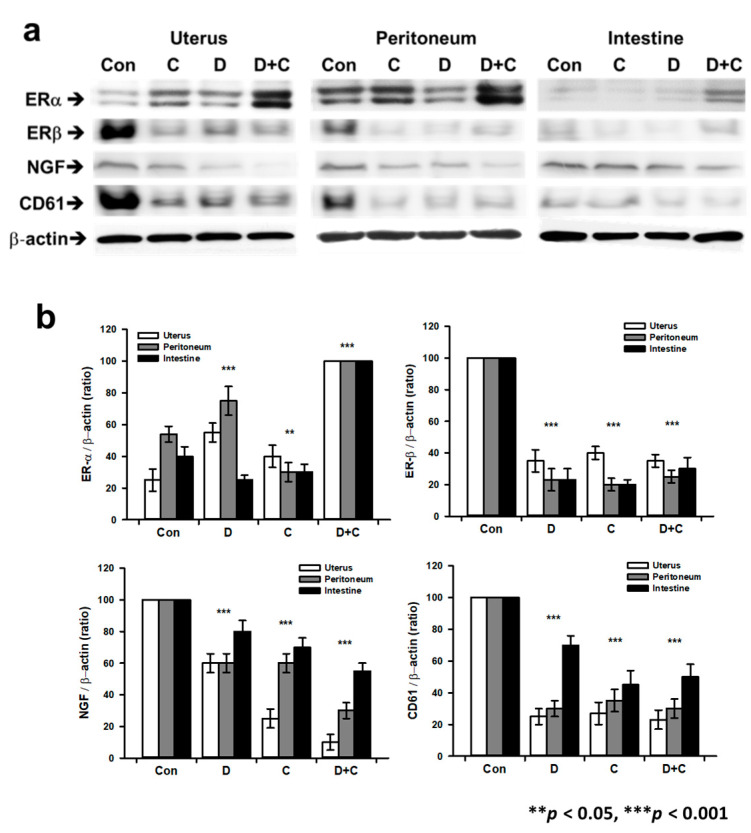
Comparative analysis of protein expression in the inflammatory lesions of endometriosis tissue using anti-ER-α, ER-β, CD61, and NGF antibodies. (**a**) Western blot images of ER-α, ER-β, CD61, and NGF protein expression patterns in the control and experimental groups (Dienogest, Cabergoline, and combined treatment). (**b**) Graph of Western blot band intensity of the control and experimental groups according to medication. All experiments were repeated three times for statistical analysis (±SD, ** *p* < 0.01 and *** *p* < 0.001).

Figure 5 shows quantitative analysis at the protein level by Western blotting. We analyzed endometriosis- and inflammation-related proteins, such as ER-α, ER-β, CD61, and NGF, in the peritoneum, uterus, and intestine of endometriosis samples. First, the ER-α level was significantly increased in group D + C compared to the control (F 21.396). ER-α levels were higher in group D + C than in group D, C. ER-β levels were significantly decreased in groups D, C, and D + C compared to the control (F 3.335). NGF levels were also decreased in groups D, C, and D + C compared to the control, with group D + C showing a larger decrease than group D or C (F 17.516). CD61 expression showed similar patterns as NGF in all medication groups (F 9.805).

## 4. Discussion

In this study, we developed an endometriosis therapeutic model using laboratory animals and tested new therapeutic medicines. We found that Cabergoline is as effective as Dienogest for endometriosis treatment, and their combination has a stronger effect than the individual medications.

This endometriosis animal model showed a human endometriosis phenotype, such as increased expression levels of Er-β, Ccl2, Ccl5, and Il-6. The chronic pelvic pain marker NGF also showed significantly higher expression in endometriosis-induced animals. We then found that new medications, such as Cabergoline, exerted therapeutic effects on endometriosis, similar to Dienogest. Dienogest is a routine medication for endometriosis treatment. In one retrospective study, the patients with endometriosis who had failed a previous IVF (In vitro fertilization) cycle received 3 months of treatment with Dienogest (2 mg/daily) before the next IVF cycle. The cumulative implantation, clinical pregnancy, and live birth rates were significantly higher in the Dienogest-treated than in the non-treated group [39]. However, it cannot be applied during pregnancy or in people who are preparing for pregnancy [40]. On the other hand, Cabergoline is a medication that is safe for pregnancy and acceptable for endometriosis treatment during ART and in pregnant women [34,41]. Endometriosis patients who are preparing for pregnancy want to be treated for endometriosis and undergo ART at the same time. Therefore, new functional drugs, such as dopamine agonists, need to be developed for endometriosis therapy to treat patients during ART.

First, we successfully prepared an endometriosis animal model by intraperitoneally injecting endometrial tissue into recipient mice. Endometriosis-induced mice clearly exhibited lesions similar to human peritoneal lesions with respect to ER expression, inflammation, and macrophage infiltration, providing an opportunity for new drug studies. ER-α and beta ER-β are nuclear transcription factors and regulators of several physiological processes in humans like cancer, osteoporosis, metabolic homeostatic, cardiovascular diseases, neurodegeneration, and inflammation [42,43]. In the immunohistochemistry study, the expression pattern and localization of inflammation-related proteins, such as ER-β and NGF, were found to be decreased after treatment with Dienogest or Cabergoline, showing a greater decrease in the group treated with combined medications than in the group treated with a single medication. For those with endometriosis symptoms, ER-α has significantly lower expression than ER-β in inflammatory lesions [44]. The high level of ER-beta in the endometriotic stromal cells may suppress progesterone receptor expression and enhance the cyclo-oxygenase 2 level, which is associated with progesterone resistance and inflammation [45]. Previous studies have reported that ER-β is a major target molecule to develop therapeutic medications for endometriosis [44]. In gene and protein analysis of ER-α and ER-β, the Cabergoline-treated group showed significant suppression of ER-β, with elevation of ER-α. This means that Cabergoline alone has a therapeutic effect on the recovery of inflammatory lesions. ER-β expression was also significantly decreased in the group treated with both medications compared to the control group.

Recent studies have also revealed that NGF is associated with the mechanism underlying pain in endometriosis [18,46]. NGF is highly expressed in the peritoneal fluid, ovary, and uterus of endometriosis patients [47]. NGF can function as a pain mediator. It is believed to be directly involved in hyperalgesia and persistent inflammatory pain [46]. Mice with endometriosis showed high expression levels of NGF in lesion tissues of the peritoneum, uterus, and intestine. NGF expression was found to be significantly decreased in all medication-treated groups. In particular, the group treated with a combination of Dienogest and Cabergoline showed a higher therapeutic effect on endometriosis than the single medication group. Compared to the control group, all groups treated with medications also showed a therapeutic effect, as indicated by the inflammation indicator CD61.

All drug treatments showed therapeutic effects on gene expression in endometriosis tissues. ER-β expression was decreased in the peritoneum, uterus, and intestine after treatment with the medications (alone or in combination). ER-α showed a slight increase in expression in all groups treated with the medications. All inflammation-related genes, such as Ccl2, Ccl5, and Il-6, showed the synergistic effect of the combined treatment, consistent with previous studies [48,49]. The inflammation-related gene expression profile showed that Cabergoline had a therapeutic effect similar to that of Dienogest.

Therapeutic drugs, such as combined medications, had a synergic effect on endometriosis compared to the individual medications, and their combination promoted strong recovery of peritoneum, uterus, and intestine lesions in the endometriosis animal model. Therefore, Cabergoline could be used as an alternative to Dienogest during ART for patients with infertility. Additionally, both medications dramatically decreased NGF expression compared to that of the positive control group. Thus, both medications could improve the quality of life of patients by decreasing their chronic pelvic pain. Importantly, this study showed that the combined (Dienogest + Cabergoline) medications have a synergistic effect on endometriosis treatment compared to the individual medications.

We established an animal model of endometriosis mimicking human endometriosis. Then, we tested the efficacy of dopamine agonists for endometriosis using this model. Our results indicate that Cabergoline had a similar therapeutic effect to Dienogest, that Cabergoline could be used as an alternative to Dienogest during ART for patients with infertility, and that the combined (Dienogest + Cabergoline) medications have a synergistic effect on endometriosis compared to the individual medications. Furthermore, novel drug delivery methods like nanotechnologies will be able to target the drug at the disease level, thus improving the efficacy and compliance of available treatments and development of alternative medical approaches [50].

## Figures and Tables

**Table 1:** Primer sequences for gene amplification by RT-PCR (real-time polymerase chain reaction).

Gene	Forward Primer	Reverse Primer
Er-α	TCC CTG ATG TCA ATT GCC CT	ATG GTG GGA TCT GTG AGG TG
Er-β	GCC AAC CTC CTG ATG CTT CT	TGT GAC TGG AGG TTC TGG GA
Ccl2	AGC CAA CTC TCA CTG AAG CC	GGA CCC ATT CCT TCT TGG GG
Ccl5	CAA GTG TGT GCC AAC CCA GA	CAA GCT GGC TAG GAC TAG AGC
Il-6	CCC CAA TTT CCA ATG CTC TCC	CGC ACT AGG TTT GCC GAG TA
Gapdh	TGT GAA CGG ATT TGG CCG TA	ACT GTG CCG TTG AAT TTG CC

**Table 2:** The number of inflammatory lesions positive and no inflammatory mice ratios.

	Positive Inflammatory Lesions Mice Ratio (%)	No Inflammatory Mice Ration (%)
Control	93.33 *	6.66
Dienogest	6.66 *	93.33
Cabergoline	0 *	100
Dienogest + Cabergoline	0 *	100

* *p* < 0.001.
